# Developmental and Evolutionary Significance of the Zygomatic Bone

**DOI:** 10.1002/ar.23449

**Published:** 2016-11-15

**Authors:** Yann Heuzé, Kazuhiko Kawasaki, Tobias Schwarz, Jeffrey J. Schoenebeck, Joan T. Richtsmeier

**Affiliations:** ^1^ UMR5199 PACEA, Bordeaux Archaeological Sciences Cluster of Excellence, Université De Bordeaux; ^2^ Department of Anthropology, Pennsylvania State University, University Park, PA; ^3^ Department of Veterinary Clinical Studies, Royal (Dick) School of Veterinary StudiesUniversity of Edinburgh, Easter Bush Veterinary Centre, RoslinMidlothianUK; ^4^ Division of Genetics and Genomics, The Roslin Institute and Royal (Dick) School of Veterinary StudiesUniversity of Edinburgh, Easter BushMidlothianUK

**Keywords:** midfacial retrusion, midfacial hypoplasia, prognathism, FGFR‐related craniosynostosis syndromes, domesticated dogs, New World monkeys, Old World monkeys

## Abstract

The zygomatic bone is derived evolutionarily from the orbital series. In most modern mammals the zygomatic bone forms a large part of the face and usually serves as a bridge that connects the facial skeleton to the neurocranium. Our aim is to provide information on the contribution of the zygomatic bone to variation in midfacial protrusion using three samples; humans, domesticated dogs, and monkeys. In each case, variation in midface protrusion is a heritable trait produced by one of three classes of transmission: localized dysmorphology associated with single gene dysfunction, selective breeding, or long‐term evolution from a common ancestor. We hypothesize that the shape of the zygomatic bone reflects its role in stabilizing the connection between facial skeleton and neurocranium and consequently, changes in facial protrusion are more strongly reflected by the maxilla and premaxilla. Our geometric morphometric analyses support our hypothesis suggesting that the shape of the zygomatic bone has less to do with facial protrusion. By morphometrically dissecting the zygomatic bone we have determined a degree of modularity among parts of the midfacial skeleton suggesting that these components have the ability to vary independently and thus can evolve differentially. From these purely morphometric data, we propose that the neural crest cells that are fated to contribute to the zygomatic bone experience developmental cues that distinguish them from the maxilla and premaxilla. The spatiotemporal and molecular identity of the cues that impart zygoma progenitors with their identity remains an open question that will require alternative data sets. Anat Rec, 299:1616–1630, 2016. © 2016 The Authors The Anatomical Record Published by Wiley Periodicals, Inc.

## INTRODUCTION

“If these generalizations are meaningful we may suspect that the establishment of the jugal's squamosal connection, together with the elimination of the constraining connection with the quadratojugal, created a genetic condition in which the jugal had a lot of freedom.” (http://palaeos.com/vertebrates/bones/dermal/orbital-jugal2.html)

The facial skeleton is a complex functioning unit that aids in the support, protection and integration of the systems responsible for neural processing, vision, olfaction, hearing, feeding, respiration, and vocalization. The facial skeleton varies markedly in morphology across extant vertebrate species, and changes are apparent over evolutionary time as some of the most important aspects of vertebrate adaptations involve changes in facial morphology.

Bones of the craniofacial skeleton, whether derived from neural crest or mesoderm and whether formed intramembranously or endochondrally begin as a group of mesenchymal cells that interact with an epithelia, form a condensation, and then differentiate along either a chondrogenic or osteogenic path (Hall and Miyake, 2000). The mineralization of collagen in the matrix secreted by osteoblasts and the consequent formation of bone is under the control of genes organized into networks (Long, 2012). In addition to influences from genetic signaling, dynamic physical interactions among soft and hard tissues contribute to the generation of complex shapes of facial bones such that the head forms as a set of interacting components contributing to an integrated whole (Martínez‐Abadías et al., 2013b; Lee et al., 2015).

The zygomatic bone is part of the facial skeleton of mammals, most reptiles, amphibians, and birds, but is absent in living amphibians. In reptiles (excepting turtles), the zygomatic bone forms a relatively narrow bar separating the orbit from the inferior temporal fossa. The bone is similarly reduced in birds. Articulation of the zygomatic bone with the squamosal forms the zygomatic arch that serves as the lateral boundary of the temporal fossa. In non‐mammalian species that have no zygomatic arch, the zygomatic bone is called the jugal (de Beer, 1936; Kardong, 2011).

Evolutionary changes in the vertebrate facial skeleton are thought to reflect functional and structural changes coincident with an expanding dietary repertoire that included mobile predation and necessitated variation in tooth size, dental formulae, muscle attachments, and relative positioning of the major sense organs (Kardong, 2011). The evolution of higher vertebrates (reptiles, birds, mammals) required changes in developmental patterning that allowed greater variation in jaw morphology affecting individual bones of the facial skeleton. This variation served as the raw material for the evolution of a diverse collection of facial forms of varied widths and degrees of projection and an equally diverse assortment of zygomatic morphologies. These variants precipitated modifications favored by selection or by genetic drift that became established through changes in development under the control of genes.

The skull of mammals represents a highly modified synapsid pattern (Moore, 1981; Kardong, 2011). Many of the diagnostic characteristics of mammals (e.g., young that suckle, enlarged brain, single dentary, akinetic skull) are tied closely to the craniofacial skeleton (Kemp, 2005). Among mammals, the zygomatic bone varies markedly not just between monotremes, marsupials and eutherians but also within eutherians. In most mammals, the zygomatic bone has a malar (facial) surface and a temporal surface and connects the facial skeleton (usually via the maxillae) with the neurocranium at one or more points. Among eutherians, the zygomatic bone can form a large portion of the bony orbit while simultaneously contributing considerably to the malar surface (as in primates and canids), but it may also be reduced to a small peg within the zygomatic arch (as in mice and rabbits). In those cases where the zygomatic bone contributes to the bony orbit, processes of the zygomatic and frontal bones articulate to form the postorbital bar that encompasses the lateral aspect of the eye separating the temporal fossa from the globe of the eye. Complete postorbital bars have evolved convergently in several mammalian clades and the current explanation for this bony partition is that it evolved to insulate the globe of the eye and other orbital contents from mechanical disturbance by the anterior temporalis muscle during mastication (Cartmill, 1980; Ross and Hylander, 2000; Menegaz and Kirk, 2009).

Although the ‘insulation hypothesis” (Cartmill, 1980) provides a functional explanation for changes in the orbital surface and orbital process of the zygomatic bone that occurred in concert with changing masticatory demands, equally profound changes have occurred in the malar part of the zygomatic bone. Remarkably, the contribution of the zygomatic bone to variation in midfacial prognathism has not been extensively studied. Here we present a short synopsis of evolution and development of the zygomatic bone to provide information on which we base our expectations regarding the contribution of the malar portion of the zygomatic bone to variation in midfacial protrusion. This is followed by three comparative studies of zygomatic bone shape in extant eutherian species. In each case, variation in midfacial protrusion is tied to one of three mechanisms: genetic mutation, selective breeding, or long term evolution from a common ancestor.

### Evolution of the Zygomatic Bone: A Single Bone Within a Complex System

The skull is a particularly instructive example of a complex skeletal system that has been classified in diverse ways to enable the people who study skulls to make sense of them (Hall, 2014). The skull represents the integration of two skeletal systems: the endoskeleton that develops internally as cartilage (phylogenetically and ontogenetically) and the exoskeleton (the dermal skeleton) that ossifies as dermal bone (Kawasaki and Richtsmeier, In press; Hall and Miyake, 2000; Hall, 2014, 2015). The cranial portion of the endoskeleton is composed of two major structures: the chondrocranium that is composed of cartilage and cartilage bone and supports the brain and other cranial sense organs, and the pharyngeal skeleton that forms much of the jaws and structures of the neck in modern vertebrates. The dermal cranial skeleton, referred to as the dermatocranium, almost completely covers components of the cranial endoskeleton and replaces them as development proceeds.

The modern vertebrate skull is a composite structure that evolved from the interaction and eventual integration of the cranial endoskeleton and the dermatocranium. The pharyngeal skeleton (variously called the splanchnocranium, viscerocranium, and visceral skeleton) is the most ancient component of the endoskeleton and arose as cartilaginous gill bars that reinforced the pharyngeal slits of protochordates (Kardong, 2011). The chondrocranium was the next part of the cranial endoskeleton to arise and formed as separate cartilages underneath and surrounding the brain and other cranial soft tissue organs (Kawasaki and Richtsmeier, in press). The final part of the skull to appear evolutionarily was the dermatocranium, originally an all‐encompassing bony “shield” composed of several dermal series, each comprised of multiple bones, that completed the skull and functioned as external armor essentially covering the chondrocranium and the pharyngeal skeleton (Janvier, 1996; Donoghue and Keating, 2014). The zygomatic bone in modern vertebrates derives from the jugal that arose evolutionarily as an element of the “orbital” (or circumorbital) series of the dermatocranium (Gregory, 1929; Romer, 1977; Kardong, 2011). The orbital series consists of a group of specialized dermal bones located around and behind the globe of the eye that served to protect the eye and the anterior part of the upper jaw from damage or deformation. Evolutionary observations of bony vertebrates place the jugal in articulation with the lacrimal anteriorly (Goodrich, 1930; Schultze, 1993). A consistent articulation between the jugal and the squamosal is established by the time of the Therapsida. Evolutionary descriptions of the jugal demonstrate its expansion along with the elongation of the jaw and enlargement of the postorbital skull during tetrapod evolution (Anon).

**Figure 1:**
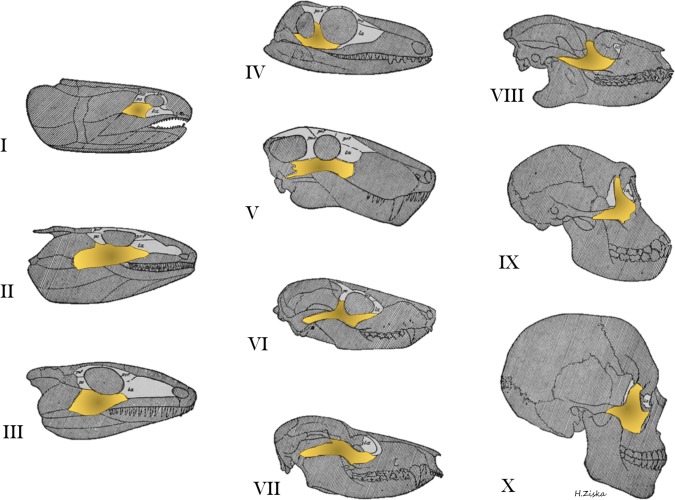
Evolution of circumorbital bones of the dermatocranium from William King Gregory's, “Our face from fish to man” (1929) showing the change in zygomatic morphology over evolutionary time. The orbital series is figured in light gray while the zygoma or jugal is highlighted in yellow. I, Lobe‐finned fish, Devonian age; II, Primitive amphibian, Lower Carboniferous; III, Primitive cotylosaurian reptile, Permo‐Carboniferous; IV, Primitive theromorph reptile, Permo‐Carboniferous; V, Gorgonopsian reptile, Permian; VI, Primitive cynodont reptile, Triassic; VII, Primitive marsupial, Upper Cretaceous; VIII, Primitive primate, Eocene; IX, Anthropoid (female chimpanzee), Recent; Man, Recent. Gregory was an expert primatologist, paleontologist, and functional and comparative morphologist and a leading contributor to several theories of evolution including the “Palimpsest theory” (Gregory, 1947) and “Williston's Law” (Gregory, 1935). Adapted from Gregory's (1929) Figure 51, p.81.

William King Gregory (Gregory, 1929) summarized the evolution of the jugal from the orbital series of the dermatocranium using evidence from fossil specimens and revealed that as previously separate parts of the dermatocranium coalesced and others dropped out, the jugal took on different shapes (Fig. 1). Though Gregory's ideas about evolution were presented more in the form of an anatomical ladder of progress than an evolutionary tree, and the details of jugal evolution has far more twists and turns than what is depicted in Figure 1, Gregory's work provides a simple summary that can serve as the basis for understanding the evolution of the zygomatic bone from a member of the circumorbital series in lobe finned fishes through the elimination of its connection with the quadratojugal to its stable connection with the squamosal (temporal bone) in marsupials and anthropoids. The evolutionary hypothesis depicted in these diagrams has not changed significantly in modern treatises (Janvier, 1996; Kardong, 2011) and remains compelling today.

### Development of the Zygomatic Bone in Modern Vertebrates

The evolution of the vertebrate head was made possible in part by the evolution of a novel cell population: the neural crest (Gans and Northcutt, 1983; Hall, 1999; Buitrago‐Delgado et al., 2015). Neural crest cells are specific to vertebrates and delaminate from the ectoderm of the closing neural tube and migrate to diverse locations including formative cranial structures (i.e., the pharyngeal arches and various facial prominences) where they differentiate to form a multitude of tissues including cartilage and bone. Importantly, not all cranial cartilage and bone in vertebrates derives from cranial neural crest but can also derive from paraxial mesoderm. Whether specific cranial bones are derived from neural crest or mesoderm has been shown to vary across vertebrate model organisms (Jiang et al., 2002; Gross and Hanken, 2008; Piekarski et al., 2014) and this variation likely exists across non‐model organisms too.

Though direct experimental evidence is scarce, it appears likely that the neural crest cells responsible for the formation of the zygomatic bone in mammals come from the streams of neural crest cells that populate the first pharyngeal arch (Lee et al., 2004; Gross and Hanken, 2008). Bones derived from neural crest cells that populate this embryonic facial structure form the bony nasal passages, palate and external facial surface and include: the mandible, maxilla, incus, malleus, zygomatic, palatine (and potentially part of the temporal). Changes in the shapes and sizes of these bones contribute to variation in facial morphology.

The earliest skeleton of developing modern vertebrates is cartilaginous, consisting primarily of the post cranial endoskeleton and the two cranial components of the endoskeleton: the pharyngeal skeleton and the chondrocranium (Kawasaki and Richtsmeier, in press). As prenatal development proceeds, bones of the dermatocranium (the cranial component of the dermal or exo‐ skeleton) begin to form by intramembranous ossification, giving rise to bones of the cranial vault and facial skeleton (including the zygomatic). Simultaneously, portions of the chondrocranium begin endochondral ossification while other chondrocranial elements disappear (Kawasaki and Richtsmeier, in press). By birth, the skull is an amalgam of elements derived from the endoskeleton and dermal skeleton.

**Figure 2:**
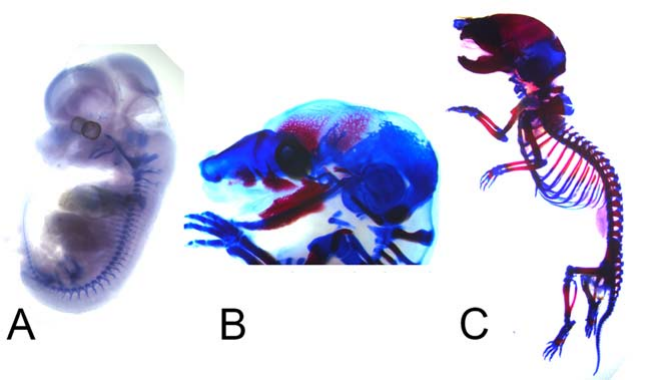
Mouse (*Mus musculus*) skeletal development at three distinct developmental stages: **A**, Embryonic day 12.5 (E12.5); **B**, E15; **C**, postnatal day 0 (P0). Alizarin red (bone) and alcian blue (cartilage) staining was used at these three developmental stages.

Although we are currently gaining new information from additional model organisms, the animal that is most frequently used in laboratory research to inform our understanding of human skeletal development is the mouse (*Mus musculus*). The earliest skeleton of the developing mouse is visible at approximately embryonic day 12.5 (E12.5). The earliest skeleton is cartilaginous and consists primarily of the post cranial skeleton, although Meckel's cartilage is clearly visible (Fig. 2a). By E15, some of the postcranial skeleton is undergoing endochondral ossification and bones of the dermal skeleton are developing intramembranously including the more rostral bones of the cranial vault, the jaws and other facial bones including the zygomatic (Fig. 2b). By birth, the skeleton is a composite of still‐cartilaginous endoskeletal structures along with ossified portions of the endoskeleton and dermal skeleton (Fig. 2c).

### Problem Formulation

As we have noted, the mammalian craniofacial skeleton represents an assemblage of skeletal elements with diverse evolutionary and developmental origins that form to enclose the brain, the primary sensory organs, and the oral and respiratory cavities. Embryologically, the pharyngeal skeleton and chondrocranium are the earliest structures of the skull to appear. Some elements of the chondrocranium undergo endochondral ossification, some regress, and others remain as cartilage. The dermatocranium forms a bit later through intramembranous ossification over the surfaces of many elements of the pharyngeal skeleton and chondrocranium. Given the complex architecture of cranial soft tissues and of the skull that supports and protects them, the genetic and developmental links among these tissues must have been robust, but labile providing the ability to vary, and over time, evolve. Evolutionary changes in vertebrate craniofacial morphology would require precisely coordinated modification among soft tissues and the various craniofacial skeletal components. Transformations of the craniofacial skeleton occurring over shorter time scales would require similar synchronization. For example, one of the major changes in the primate facial skeleton that has repeatedly appeared or conversely been lost in evolution is facial retrusion, or its opposite, prognathism. Variation in skull morphology variation among modern dog breeds including muzzle morphology, is in large part a human‐created phenomenon, generated rapidly over a relatively short period of time through artificial selection (Schoenebeck et al., 2012). Finally, genetic mutations responsible for some human craniofacial disorders cause midfacial retrusion (hypoplasia), a condition that is not only exceedingly common but also particularly difficult to manage clinically (Cunningham et al., 2007), affecting not only the size but also the shape of the facial skeleton. Midfacial retrusion is defined as the “posterior positioning and/or vertical shortening of the infraorbital and perialar regions, or increased concavity of the face and/or reduced nasolabial angle” (Allanson et al., 2009), a definition which makes midfacial retrusion or hypoplasia a catch‐all diagnosis in medicine and a vague trait in evolutionary studies. This definition confirms the developmental and morphological complexity of this phenotypic trait that is not only expressed in the anteroposterior dimension but in the two other dimensions as well. The same complexity accompanies the opposite phenotype, which is midfacial protrusion. Regardless of the source of the variation, changes in midfacial architecture require a coordinated modification of individual bones as well as any necessary adjustments in the articulation of the facial skeleton with the neurocranium.

Because among mammals the common pattern is for the zygomatic bone to serve as the physical bridge that connects the facial skeleton with the neurocranium, we hypothesize that the shape of the zygomatic bone reflects its role in stabilizing this connection and that changes in facial prognathism (or its opposite retrusion) are more strongly reflected in those facial bones situated rostral to the zygomatic (maxilla, premaxilla). To explore this hypothesis, we present three comparative analyses of zygomatic bone variation in samples that differ in the degree of facial prognathism.

#### Study 1. Facial retrusion in craniosynostosis

First we compare the general facial shape and specific zygomatic shape in typically developing children and children who carry mutations known to cause craniosynostosis syndromes. Each of the craniosynostosis syndromes considered here is caused by a mutation in one of the genes coding for fibroblast growth factor receptors (FGFRs), and are collectively referred to as the FGFR‐related craniosynostosis syndromes (Wilkie et al., 2010, 2002). Typically, the coronal suture is prematurely fused either bilaterally or unilaterally in these syndromes and the face is dysmorphic and retruded, a condition commonly referred to as “midfacial hypoplasia,” or facial retrusion (Heuzé et al., 2014).

Fibroblast growth factors (FGFs) are secreted molecules that signal through the activation of their cognate tyrosine kinase receptors, the fibroblast growth factor receptors, or FGFRs. The FGFR mutations associated with craniosynostosis syndromes either alter the binding affinity for specific FGFRs toward all or a particular subset of FGFs (Anderson et al., 1998; Ibrahimi et al., 2001), or cause constitutive activation of the FGFR pathway stimulating FGFR dimerization and intracellular activity without binding of an FGF ligand (Galvin et al., 1996; Mai et al., 2010). For cells that constitute the coronal suture mesenchyme, the downstream consequence of these *FGFR* mutations is the onset of runt‐related transcription factor 2 (*RUNX2*) expressions, essential for the differentiation of osteoblasts. Expression of *RUNX2* leads suture mesenchyme cells to differentiate into osteoblasts that deposit bone and eventually unify the two osteogenic fronts of the suture (Komori, 2011; Maeno et al., 2011). In mouse models for the FGFR‐related craniosynostosis syndromes premature closure of a cranial vault suture is precipitated by changes in cell behaviors including a diminished population of undifferentiated mesenchymal cells and inappropriate or premature differentiation of precursor cells into osteoblasts (Holmes et al., 2009; Deckelbaum et al., 2012) that can also be associated with diminished cranial bone mineralization (Twigg et al., 2009; Percival et al., 2012; Liu et al., 2013).

Though much research has focused on cranial vault suture closure in these syndromes, we have shown in humans and mouse models for these conditions that dysmorphogenesis is more severe in the facial skeleton relative to the cranial vault and that distinct facial phenotypes and patterns of variation exist for each diagnostic group (Martínez‐Abadías et al., 2010, 2013a; b; Heuzé et al., 2014; Motch Perrine et al., 2014). Here we focus specifically on the changes in facial shape in human infants diagnosed with these syndromes.

#### Study 2. Facial retrusion in domestic dogs

**Figure 3:**
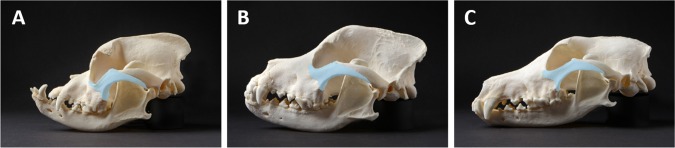
Lateral view of canine skulls illustrates the shape and size variation of the zygoma (pseudo‐colored blue): **A**, boxer; **B**, Rottweiler; **C**, greyhound.

The second example compares breeds of dogs that vary in skull shape as a result of artificial selection and consolidation of desired traits (selective breeding). Coupling genetic profiles with museum specimen measurements, Schoenebeck et al. used genome wide association to identify five genetic loci that are responsible for the craniofacial differences found in dog breeds that fall into two generalized skull shapes: brachycephaly and dolichocephaly (Schoenebeck et al., 2012). In humans these terms are used to describe a disproportionately short, broad head as determined by the cephalic index, which is the ratio of the maximum width of the head multiplied by 100 divided by its maximum length. Head width and length in humans are typically measured on the cranial vault bones and do not include any facial structures. In dogs, these terms include a consideration of the facial profile, or muzzle prognathism, so that breeds like the pug and bulldog typify brachycephalic dogs while the collie and Afghan hound represent dolichocephaly in dogs. Here we consider a large number of dog breeds to determine how zygomatic shape contributes to canine facial prognathism (Fig. 3).

#### Study 3. Facial retrusion in new world monkeys

Finally, we compare facial shape in species representing two large, geographically separated, monophyletic primate groups: the Platyrrhini (New World monkeys) and Catarrhini (Old World monkeys). The phylogenetic separation of these two groups dates to ∼35–40 million years ago by fossil and mitochondrial genomic evidence (Fleagle, 2000; Schrago and Russo, 2003). Morphologically these groups differ in certain craniofacial characteristics. New World monkeys have three premolars, whereas Old World anthropoids have two, and the bony structure of the inner ear differs between the two groups. Principally though, Old World monkeys tend to have relatively elongated muzzles and raised noses, whereas New World monkeys have flatter muzzles with nostrils that face laterally (Napier and Napier, 1985) such that the two groups differ in degree of facial projection (or retrusion).

Using these samples, we conducted three comparative analyses to answer the question: How does the zygomatic bone contribute to variation in facial prognathism?

## MATERIALS AND METHODS

### Data

Data used here represent three dimensional coordinates of biologically relevant landmarks located on the skulls of human infants, on the skulls of a collection of modern dog breeds, and on the skulls of four species of primates. As the data were acquired for previous studies, the specific landmarks used vary across the three case studies and the three dimensional coordinates of landmark locations were collected using different methods for each study as summarized briefly below.

#### Human infants

As part of a previous study of facial morphology in craniosynostosis syndromes (Heuzé et al., 2014), we amassed preoperative computed tomography (CT) images of children aged 0–23 months diagnosed with FGFR‐related craniosynostosis syndromes that were acquired by several medical centers in France, USA, Taiwan, and Spain over the past 10 years. Use of the CT images was approved by the Institutional Review Boards of the Pennsylvania State University and the participating institutions and the images were acquired in accordance with institutional guidelines. All collected images were anonymized and no information other than sex, age at the time of the CT exam, and causative mutation were available.

Our sample consists of 3D CT images of 43 individuals genetically and/or clinically diagnosed with Apert syndrome (AS, *N* = 21), Crouzon syndrome (CS; *N* = 9), Muenke syndrome (MS; N = 6), or Pfeiffer syndrome (PS, *N* = 7), and 38 unaffected individuals. In our sample, 38 of 43 craniosynostosis syndrome cases showed premature fusion of the coronal suture, while CT images revealed a lack of closure of any cranial vault suture in three CS individuals and one AS individual. The specific causative mutation in *FGFR1*, *FGFR2*, or *FGFR3* was identified in 19 cases by genetic screening while diagnosis of the remaining cases (*N* = 24) is based solely on clinical evaluation (Heuzé et al., 2014). The unaffected sample consists of images of children without premature suture closure who underwent CT scanning for conditions not associated with craniosynostosis (e.g., seizures, suspected brain anomalies).

**Figure 4:**
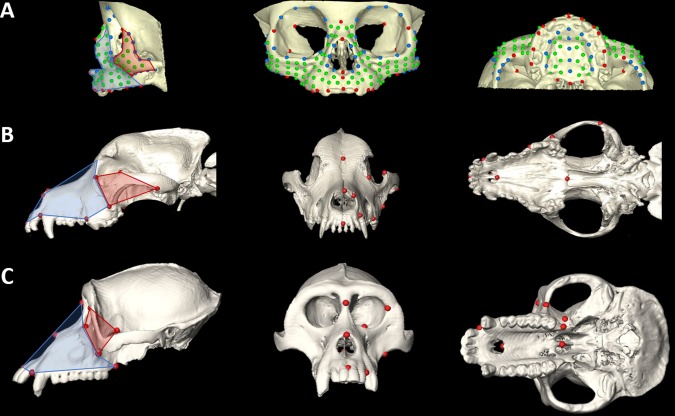
Skull reconstructions with measured landmarks (lateral, anterior, inferior views): **A**, human infant; **B**, labrador retriever; **C**, Macaca fascicularis. For more details on landmarks and semilandmarks measured on human infants see Heuzé et al. (2014). Landmarks measured on dogs include: nasion, nasale, anteriormost point on nasal bone, infradentale, premaxilla‐maxilla suture posterior to I2, first molar anteriormost point on alveolar bone, posterior nasal spine, premaxilla‐maxilla suture on hard palate, posteriormost point on zygomatic (temporal‐zygoma suture), superiormost point on zygomatic, inferiormost point on zygomatic (maxilla‐zygoma suture), zygoma‐lacrimal suture. Landmarks measured on monkeys include: nasion, nasale, infra dentale, premaxilla‐maxilla suture posterior to I2, anteriormost point on frontal‐zygoma suture, superiormost point on maxilla‐zygoma suture, inferiormost point on maxilla‐zygoma suture, maxillary tuberosity, pterygoid fossa, posterior nasal spine.

Isosurfaces of the skulls were reconstructed from the CT images using a threshold that enabled visualization of bone. A set of 39 anatomical landmarks was defined and located on the 3D reconstruction of the facial skeleton of each individual and the corresponding *x*,*y*,*z* coordinates were recorded with Avizo (Visualization Sciences Group, SAS). In addition to the anatomical landmarks, semilandmarks were defined on 8 predefined curves and two surface patches on each facial skeleton (Fig. 4a; for details see Heuzé et al., 2010, 2012). Semilandmarks present “deficient” coordinates and were slid along the curves and surface patches according to a sliding algorithm that minimizes the bending energy to define their final location on the defined curves or surfaces (Bookstein, 1997; Gunz et al., 2005). Once slid, semilandmarks acquire a geometric correspondence (not necessarily a biologic correspondence) across individuals so that comparative analyses can be conducted. The 3D coordinates of semilandmarks were computed using Viewbox 4 (dHAL software, Athens, Greece).

#### Modern dog breeds

CT images were acquired retrospectively from canine patients that presented to the Royal (Dick) School of Veterinary Studies Hospital for Small Animals for diagnostic imaging. All data are derived from adult dogs (age >24 months). Breed assignments were based on owner reports. Our use of the data was in accordance with institutional guidelines that include owner consent and School of Veterinary Medicine Ethical Review Committee approval. Landmarks were identified on the left side of the facial skeleton and their 3D locations were recorded on isosurfaces of the CT images using the same protocols described above for the human CT data (Fig. 4b). In total, CT images of 51 dogs representing 16 breeds were considered in our study.

#### Nonhuman primates

Three‐dimensional coordinates of landmark locations were recorded directly from the skulls of adult male specimens of four primate species using a 3Space digitizer (Polhemus Navigation, Colchester, VT) (Fig. 4c). Access to skeletal data was provided by the National Museum of Natural History, Smithsonian Institution. Other data recorded for each skull include the teeth that have reached full occlusion, stage of spheno‐occipital synchondrosis closure and amount of M2 wear to enable aging the specimens. For each species, crania were grouped by sex and placed into one of five dental age categories. Details pertaining to landmark data collection, sexing and aging of specimens can be found in the following publications (Corner and Richtsmeier, 1991, 1992, 1993; Richtsmeier and Lele, 1993). For this study we used skulls of adult male *Cercopithecus aethiops* (*N* = 19) and *Macaca fascicularis* (*N* = 58) to represent Old World monkeys, and *Ateles geoffroyi* (*N* = 28) and *Cebus apella* (*N* = 31) to represent New World monkeys.

### Morphometric Methods

**Figure 5:**
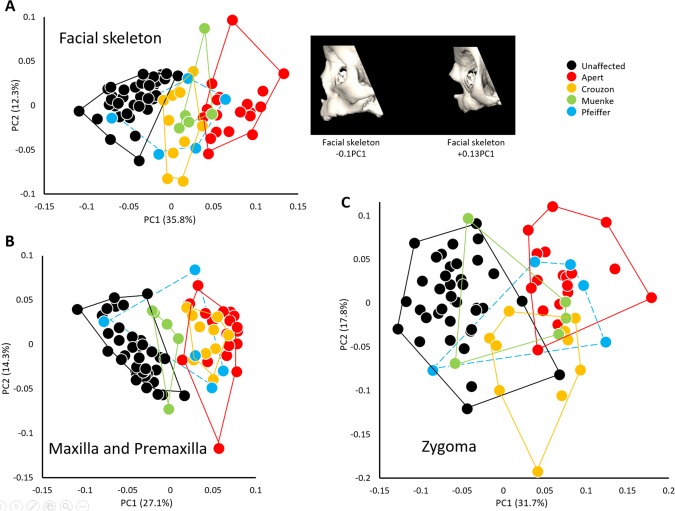
Morphological variation of the facial skeleton in infants diagnosed with FGFR‐related craniosynostosis syndromes and unaffected individuals. **A**, PCA of Procrustes shape coordinates of all landmarks and semilandmarks measured on the facial skeleton (left) and shape changes of the facial skeleton associated with the negative and positive extremes of PC1 (right); **B**, PCA of Procrustes shape coordinates of all landmarks and semilandmarks measured on the maxilla and premaxilla; **C**, PCA of Procrustes shape coordinates of all landmarks and semilandmarks measured on the zygoma.

Three separate geometric morphometric analyses were run, one per sample. Shape information for each individual/specimen defined on the basis of landmarks (and semilandmarks for humans) was extracted using general Procrustes analysis, a procedure that superimposes configurations of landmarks by shifting them to a common position, rotating, and scaling them to a standard size until a best fit of corresponding landmarks is achieved (Rohlf and Slice, 1990; Dryden and Mardia, 1998). Distinct Procrustes superimpositions were used for the analyses of specific skull anatomical units (i.e. facial skeleton; maxilla/premaxilla; zygoma, Fig. 5). The covariance matrix of the Procrustes shape coordinates was analyzed by principal components analysis (PCA) (Jolliffe, 2002) to reduce the dimensionality of the dataset. PCA transforms the original landmark coordinates into a set of new variables, the principal components (PCs), which are uncorrelated with each other. The first PC accounts for the maximum possible amount of variation and each successive PC in turn accounts for the remaining maximum possible amount of shape variation in the sample. We used PCA to determine whether the specific combination of shape variables that explain most variation is also able to successfully separate individuals into groups of known membership. The PCs contain the loadings for the linear combinations of the original variables and can be visualized as shape deformations. Because size variation is big in the dog and monkey samples, PC1 was expected to be highly correlated with size. This is in contrast to what we expect for the human sample where size variation was minimal. Geometric morphometric analyses were run in Morpho J (Klingenberg, 2011).

## RESULTS

### Zygomatic Shape in Mutation‐driven Midfacial Retrusion

The PCA analysis based on the Procrustes coordinates of the landmarks and semilandmarks that define the global facial configuration reveals three main clusters along PC1 (no significant correlation with size), which accounts for 36% of the total shape variation: unaffected individuals, patients diagnosed with Crouzon and Muenke syndrome and patients diagnosed with Apert syndrome (Fig. 5a). Patients diagnosed with Pfeiffer syndrome display a large range of shape variation overlapping with the four other groups. This arrangement of the cases along PC1 suggests that the more retrusive facial morphologies (i.e., patients diagnosed with Apert syndrome) are located at the positive extreme of PC1. A second PCA that includes only the landmarks located on the premaxilla and maxilla reveals a cluster of patients diagnosed with Apert and Crouzon syndromes on the positive end of PC1 (no significant correlation with size) and a cluster of patients diagnosed with Muenke syndrome and unaffected individuals. Patients diagnosed with Pfeiffer syndrome display a large range of shape variation as they did for the previous PCA (Fig. 5b). A third PCA that includes only the landmarks located on the zygoma does not successfully separate groups according to diagnosis with all four FGFR‐related syndromic groups overlapping with one another along PC1 (no significant correlation with size) accounting for 32% of total variation (Fig. 5c). A significant number of cases diagnosed with Crouzon and Muenke syndromes display a zygoma shape similar to that of unaffected individuals.

These results suggest that the shape of the zygoma in these syndromic cases is less characteristic of the facial morphology of the particular craniosynostosis syndromes (and by extension, the particular causative mutation). Instead, the shape of the zygomatic bone is similar across all syndromes and, in some cases overlaps with the shape of the zygomatic bone in unaffected individuals. This indicates that the zygomatic bone contributes less to the facial retrusion characteristic of craniosynostosis syndromes than the maxilla and premaxilla.

### Zygomatic Shape in Modern Dog Breeds

**Figure 6:**
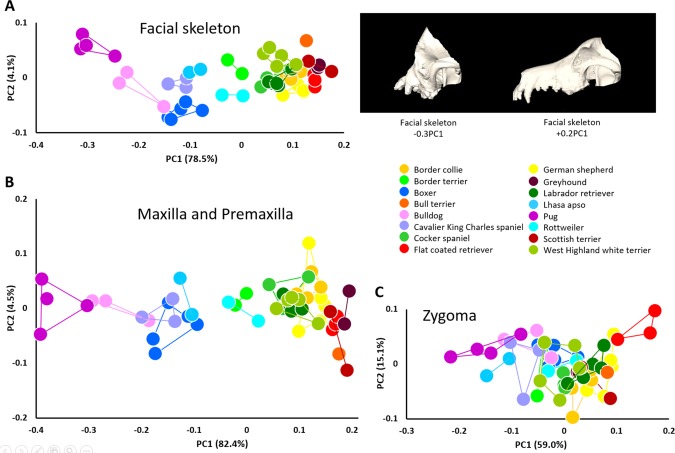
Morphological variation of the facial skeleton in modern breed dogs. **A**, PCA of all landmarks measured on the left facial skeleton (left) and shape changes of the facial skeleton associated with the negative and positive extremes of PC1 (right); **B**, PCA of all landmarks measured on the left maxilla and premaxilla; **C**, PCA of all landmarks measured on the left zygoma.

When the shape of the left canine facial skeleton is analyzed using PCA, the first PC accounts for 79% of the total shape variation for which size (CS) explains 57% (*P* < 0.0001). Shape variation of the canine facial skeleton separates canine individuals primarily along PC1 (Fig. 6a) according to the relative magnitude of facial retrusion with the pug and bulldog, and to a lesser degree Lhasa apso, cavalier King Charles spaniel, and boxer tending towards the negative end of PC1 (retrognathic dogs), and the Scottish terrier, flat coated retriever and greyhound anchoring the positive end (prognathic dogs). The border terriers and Rottweiler occupy a mid‐axis position. PCA of the maxillary and premaxillary landmarks provide a similar result with the first PC accounting for 82% of the total shape variation for which size explains 65% (*P* < 0.0001) and the breeds appearing in approximately the same order along PC1 (face vs. maxilla correlation between PC1 scores for corresponding individuals: *R* ^2^ = 0.996; *P* < 0.0001) (Fig. 6b). When only data from the zygomatic bone are analyzed by PCA the two clusters previously described with an axis whose extremes are defined by retrognathic dogs at one end and prognathic dogs at the other are no longer visible. Instead, a more homogeneous grouping appears with much increased overlap among the breeds along PC1 accounting for 59% of the total shape variation for which size explains 39% (*P* < 0.0001) (Fig. 6c). Of note, though there is considerable overlap among breeds on the basis of the analysis of zygomatic shape, the relative position of the breeds along PC1 is similar to the previous analyses with the retrognathic breeds positioned at one extreme of PC1 and the prognathic breeds at the other extreme (face vs. zygoma correlation between PC1 scores for corresponding individuals: *R* ^2^ = 0.67; *P* < 0.0001).

**Figure 7:**
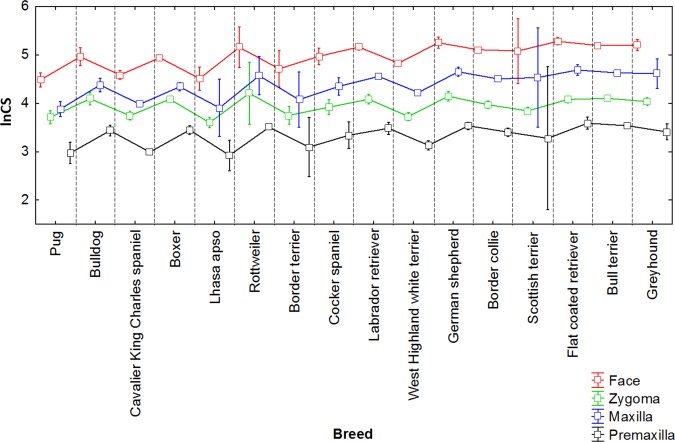
Size variation among modern breed dogs based on natural log centroid size (lnCS) for the facial skeleton, maxilla, premaxilla, and zygoma (mean; whisker, ±0.95 confidence interval).

Given the variation in size among dog breeds, we were interested in the relative size variation of the different anatomical units of the facial skeleton studied. To determine whether size varies in a pattern similar among the different anatomical units we studied (i.e., facial skeleton, premaxilla, and maxilla, zygoma), we used centroid size of the facial skeleton, premaxilla, maxilla, and zygoma as a proxy for size of each anatomical unit. The mean plot (box plot) of these four anatomical units grouped by breed (Fig. 7) indicates that size varies according to a similar pattern among the different anatomical units forming the facial skeleton. The zygoma however appears a bit “out of phase” (larger than expected) relative to the pattern of centroid size estimated for the other facial units for the retrognathic breeds (boxer, bulldog, pug and Rottweiler) and the cavalier King Charles spaniel and cocker spaniel. The large confidence intervals for the border terrier, Lhasa apso, Rottweiler, and Scottish terrier are the direct consequence of sexual dimorphism due to the fact that we only have one male and one female for these breeds. The residual estimates for each specimen (residuals of the multivariate regression of shape vs. size) were used to compute additional PCAs that represent the distribution of our canine data sets after the effects of allometry are removed. The PCAs computed using the residual estimates for each specimen provided a similar arrangement of the individuals, with the more retrognathic breeds clustering towards the positive end of PC1 (bulldog, boxer, Rottweiler, pug) (face vs. face without allometry correlation between PC1 scores for corresponding individuals: *R* ^2^ = 0.43; *P* < 0.0001).

### Zygomatic Shape in Old World and New World Monkeys

**Figure 8:**
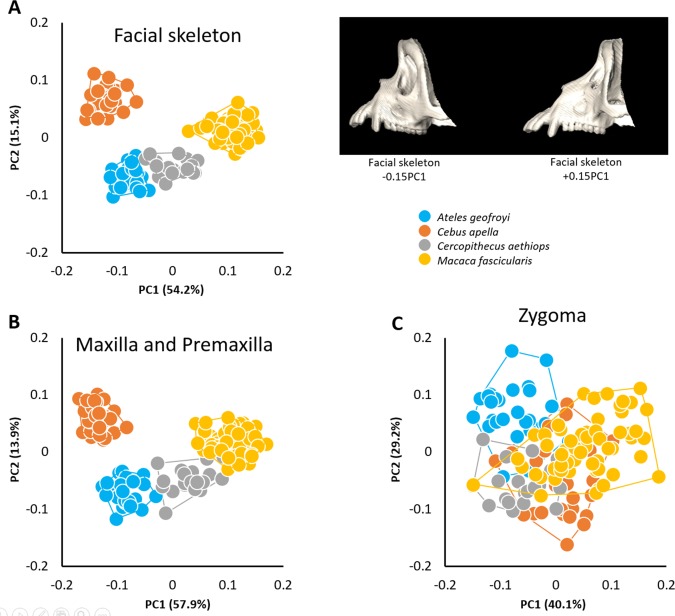
Morphological variation of the facial skeleton in Old World and New World monkeys. **A**, PCA of all landmarks measured on the left facial skeleton (left) and shape changes of the facial skeleton associated with the negative and positive extremes of PC1 (right).; **B**, PCA of all landmarks measured on the left maxilla and premaxilla; **C**, PCA of all landmarks measured on the left zygoma.

Analysis of these monkey species reveals patterns similar to those found in the analysis of humans diagnosed with craniosynostosis syndromes and of dog breeds. When all facial landmarks are used in PCA, the distribution of species‐specific facial shapes reveals no overlap when viewed across PC1 and PC2 (Fig. 8a). Species are ordered along PC1 (54% of total shape variation for which size explains 86% (*P* < 0.0001)) from least prognathic, with *Cebus apella* at the negative end of the axis to most prognathic, with *Macaca fascicularis* at the positive end. When only the landmarks located on the maxilla and premaxilla are considered, the same ordering of the four clusters appears along PC1 which accounts for 58% of total shape variation for which size explains 73% (*P* < 0.0001) (Fig. 8b). When we restrict the landmarks to only those describing zygomatic shape, the four groups overlap indicating a degree of similarity in zygoma shape across the four species (Fig. 8c) and size only explains 17% (*P* < 0.0001) of the 40% of total shape variation accounted by PC1. In these species, the degree of facial prognathism is more strongly reflected in those facial bones situated rostral to the zygomatic bone while zygomatic bone shape does not reflect the degree of facial prognathism. Our results also suggest that morphology of the zygoma in these primate species is less characteristic of the main differences between platyrrhine and catarrhine facial morphology. When allometry is removed the ordination of the four groups along PC1 is different, with *Cebus apella* still representing the least prognathic species but with *Ateles geofroyi* and *Cercopithecus aethiops* representing the most prognathic species. Consequently, the separation between platyrrhine and catarrhine is no longer evident, highlighting the role of allometry in the morphological differences between Old and New World monkeys, especially in term of prognathism.

## DISCUSSION

“Eventually, of course, all of this sudden Permo‐Triassic creativity collapses into the mundane familiarity of the mammalian zygomatic arch,…[where]…. the jugal again is forced to give up its irresponsible behavior and is harnessed to the exacting task of providing an attachment for the powerful new masseter musculature.” (http://palaeos.com/vertebrates/bones/dermal/orbital-jugal2.html)

Our study highlights commonalities in the shape variation of facial bones by quantitative analysis of the facial skeletons of placental mammals represented by humans, dogs, and monkeys. As described previously, the modern zygomatic bone evolved from the circumorbital series of bones of the dermatocranium and develops intramembranously as part of the maxillary prominence. It contributes to the facial skeleton and, along with the temporal bone, serves as part of a bony bridge that connects the facial skeleton to the neurocranium and provides attachment sites for the masseter muscle. In all three analyses, ordination along the first principal component axis of the various samples considered do not vary as much as the distance between the different groups in the shape space defined by the different PCAs corresponding to the analysis of the entire facial skeleton, the maxilla and premaxilla, and the zygoma. Indeed, the same pattern is observed: maximum distances are found among groups in the morphospace (representing shape differences) when the facial skeleton is analyzed, intermediate distances are recovered using data from the maxilla and premaxilla, and minimum distances are revealed when only the zygomatic bone is analyzed. The effect of size on shape, commonly referred to as allometry, differs depending on the groups and anatomical units considered. In the human sample, where age ranges from 0 to 23 months, allometry is minimal regardless of the anatomical unit considered. In dogs, a sample characterized by the presence of several breeds intensively selected on the basis of different traits including size, allometry explains 65, 57, and 39% of shape variation accounted by PC1 for the maxilla and premaxilla, facial skeleton, and zygoma, respectively. Finally, in monkeys, a sample composed of four different genera characterized by huge size differences, allometry explains 86, 73, and 17% of shape variation accounted by PC1 for the facial skeleton, maxilla and premaxilla, and zygoma respectively. Interestingly, the allometry effect is much smaller for the zygoma than for the maxilla and premaxilla. Importantly, even when allometry is removed, maximum distances are found among groups in the morphospace when the facial skeleton is analyzed and minimum distances are revealed when only the zygomatic bone is analyzed. Our analyses suggest that the shape of the zygomatic bone has less to do with overall facial morphology, especially prognathism, than it does with its other morphological functions: maintaining the connection between the facial skeleton and the neurocranium, providing structural support for the globe of the eye, and contributing to the skeleton of the infratemporal fossa.

Whether or not bones of the mammalian skull are of cranial neural‐crest origin has been studied by several researcher (e.g., Chai et al., 2000; Jiang et al., 2002) but the embryonic cellular origins of many of the bones of the upper jaws have not been addressed experimentally (Gross and Hanken, 2008). Though it is assumed that the bones of the facial skeleton are derived from neural crest (Jiang et al., 2002), the respective contributions of individual crest migratory streams to particular facial bones in mammals including the maxilla and zygomatic have not been evaluated (Gross and Hanken, 2008). Using observations made from numerous researchers Qiu et al. (1997) studied the expression patterns of members of the Distal‐less (*Dlx*) homeobox gene family, *Dlx1* and *Dlx2*, in the pharyngeal arches and suggested that different *Dlx* genes regulate development in different regions of the pharyngeal arches. Qiu et al. (1997) demonstrated unique and overlapping functions for these genes in patterning morphogenesis of structures derived from the proximal PA1 and PA2. The hypothesis that *Otx2* regulates the *Dlx* genes proposed by Qiu et al. (1997) was further developed by Depew and colleagues in a series of papers (Depew et al., 2002, 2005). Depew et al. (2002) investigated the mechanisms responsible for the proximodistal specification of skeletal elements within the first branchial arch. They proposed that nested *Dlx* expression patterns the arches, thereby providing cellular identity to the proximodistal axes. With the maxillary prominence considered as a part of the first pharyngeal arch (but see Lee et al., 2004), these studies provide a mechanism whereby cells within the first pharyngeal arch might establish localized subpopulations that respond differentially to molecular signals and contribute differentially to variation in facial prognathism.

In addition to patterning the skeleton and associated tissues, a molecular code may help to give cells from either the cranial or caudal portions of the first pharyngeal arch different properties such as the ability to respond to signals to differentiate, proliferate or undergo apoptosis (Lee et al., 2004). Such localized, or even nested patterns of gene expression and their potential interaction with growth factors presents a mechanism whereby specific condensations destined to become elements of the upper jaw are affected differently even though they may arise from the same migratory stream of neural crest cells. Differences in the response of cells that occupy the cranial (destined to become the maxilla) and caudal (destined to become the zygomatic and the palatine) halves of the maxillary prominence would allow for the zygomatic bone to take on a shape that does not necessarily follow the changes of the more rostral elements.

Though many bony and soft tissue anomalies contribute to the clinical problems experienced by those diagnosed with FGFR‐related craniosynostosis syndromes, the most challenging clinical manifestation of the FGFR mediated craniosynostosis may be midfacial hypoplasia (Cunningham et al., 2007). The results of our analysis suggest that: (1) the maxilla and premaxilla contribute substantially to the anatomical underpinnings of midfacial retrusion of the various craniosynostosis syndromes; (2) that the maxilla and premaxilla distinguish the midfacial morphology of Apert and Crouzon syndromes; and (3) that the zygomatic bone does not enable distinction of disease‐specific morphologies across these syndromes. Our findings provide additional evidence that midfacial retrusion (or hypoplasia), a common clinical feature of many congenital craniofacial abnormalities, consists of distinct midfacial phenotypes that, with proper analysis, might reveal which, and to what degree the various elements of the midfacial skeleton are involved. This is important as mouse genetic models in which craniofacial skeletal abnormalities are produced are analyzed according to the embryonic origins of the skeletal elements. Therefore, the anomalies collectively referred to as midfacial hypoplasia will potentially include differentially affected maxillary, premaxillary, palatine, and zygomatic bones. Our data suggests that continuing to group midfacial defects together under the catch‐all diagnosis of midfacial hypoplasia cannot aid in the identification and interpretation of phenotypes both in human and in mouse. It would be interesting to conduct a similar analysis using patients affected with Treacher Collins syndrome as the clinical definition includes a significantly reduced zygomatic bone.

Our analysis of dog skull shape again shows the zygomatic bone to be the least effective in differentiating the faces of brachycephalic and dolichocephalic dogs. While primates (and debatably humans) continue to experience purifying selection, the selective pressure placed on pedigree dogs is quite distinct. Breeders’ interest in pushing the limits of size and shape of their dogs, even at the expense of animal fitness, has resulted in an intraspecies radiation of morphological variation that continues to evolve today (Nussbaumer, 1982; Fondon and Garner, 2004; Drake and Klingenberg, 2008). The uniqueness of dog's intraspecies morphological diversity enabled us to explore the effects of both shape and size as it pertains to the facial skeleton. Like humans and primates, the shape of the canine zygoma appears resilient to shape changes in the proximodistal axis of the facial skeleton. However, when we run distinct Procrustes superimpositions for the different bones, we observe that the scaling of the maxilla and zygoma appears conversely related in the brachycephalic dogs. This observation suggests that size of the maxilla and zygoma are out of register with one another. Thus among brachycephalic dogs, the zygoma is relatively larger and the maxilla relatively smaller than one might expect from sampling non‐brachycephalic breed dogs. It is tempting to speculate that this observation is hinting at an imbalance of progenitor cells destined to contribute to formation of the maxilla and the zygoma. Broader still, perhaps the results we observe in dogs are cautioning us that substantial zygomatic variation can exist across species that present differing proximodistal facial skeleton lengths; it just happens that this variation is buried when the facial skeleton is analyzed as a whole with a unique Procrustes superimposition.

Finally, though data sets representing four species of nonhuman primates are easily distinguished using data from the face, the shape of the zygomatic bone does not differentiate these species from one another. This provides another piece of evidence that the zygomatic bone develops under a set of instructions that differentiate it from the premaxilla and maxilla with which it articulates. Results such as those published by Von Cramon‐Taubadel (2009) who reported that among different bones forming the human skull (i.e., frontal, occipital, sphenoid, temporal, parietal, maxilla, occipital) the shape of the zygomatic was the least strongly correlated with neutral genetic data, seems to support our initial hypothesis. That is, the smaller shape variation along the anteroposterior dimension of the zygomatic bone reflects its role in stabilizing the connection between the facial skeleton and the neurocranium and as such could experience stronger developmental, functional, and evolutionary constraints. We propose that the precise developmental source of the neural crest cells that contribute to the zygomatic bone and their intrinsic ability to respond or not respond to genetic signals provide the mechanism for the observed differences between the zygoma and other bones of the facial skeleton.

Morphological integration refers to the cohesion among traits in an organism that could bias the direction and rate of morphological change, so that estimation of patterns and magnitudes of morphological integration and modularity and their consequences on development are central to understanding how complex traits evolve (Wagner et al., 2007; Klingenberg, 2008; Porto et al., 2008; Koyabu et al., 2014). Our analysis of skeletal elements classified as contributing to the midface shows a subtle tendency toward modularity that might not have been recognized if the midfacial skeleton were analyzed as a single complex trait. We recognize that morphological integration is hierarchical however and surely the midface is more strongly, internally integrated than the midface is with the neurocranium, for example. However, by morphometrically dissecting the zygomatic bone from the midface we have determined a degree of modularity among parts of the midfacial skeleton suggesting that these components have the ability to vary independently and thus can evolve somewhat independently.

We have provided a description of the evolutionary and developmental sources of the zygomatic bone and demonstrated the usefulness of an approach that clearly demarcates between the contribution of the zygomatic bone and other midfacial elements to the morphology of the midfacial skeleton, components of the dermatocranium that evolved together. Measuring the degree to which the zygomatic bone contributes to midfacial prognathism enabled the formulation of ideas regarding how the zygomatic bone develops under a set of rules separate from those that govern maxillary and premaxillary development. When validated, our observations coupled with greater knowledge of zygomatic morphogenesis might further our understanding of the role of the zygomatic bone in midfacial morphology, evolution and disease.
